# Pacemaker syndrome: Thinking beyond atrioventricular dyssynchrony

**DOI:** 10.1016/j.hrcr.2024.01.012

**Published:** 2024-02-04

**Authors:** Gabriel E. Soto

**Affiliations:** Missouri Baptist Medical Center, St. Louis, Missouri

**Keywords:** Cardiac pacemaker, Diastolic heart failure, Heart failure with preserved ejection fraction, LV dyssynchrony, Cardiac physiologic pacing, Cardiac resynchronization therapy, Pacemaker syndrome

**Key Teaching Points**

• Pacemaker-syndrome is a well-recognized—if not underdiagnosed—complication of cardiac pacing typically ascribed to suboptimal atrioventricular (AV) synchrony.
• Isolated left ventricular (LV) dyssynchrony may contribute to acute symptoms typically ascribed to pacemaker syndrome even in the presence of AV synchrony and a normal left ventricular ejection fraction.
• Cardiac physiologic pacing may be a potentially useful treatment option in a subset of patients with symptomatic right ventricular pacing–induced LV dyssynchrony documented by speckle-tracking strain imaging.

## Introduction

Pacemaker syndrome has historically been used to describe a constellation of symptoms resulting from the loss of atrioventricular (AV) synchrony or retrograde conduction.^1^ Originally thought to be a problem limited to single-chamber pacemakers, it is now well established that pacemaker syndrome is not unique to single-chamber pacing and may occur in various pacing modalities.^2^ Most discussions on pacemaker syndrome ultimately attribute it to a disruption of physiologic AV synchronous contractions.

Conversely, the long-term adverse effects of chronic right ventricular (RV) pacing are well established^3^^,^^4^; however, RV pacing in and of itself has generally not been seen as a cause of the type of acute symptoms typically ascribed to pacemaker syndrome in the absence of overt loss of AV synchrony, despite studies demonstrating the potential adverse acute hemodynamic effects of RV-only pacing.^5^, ^6^, ^7^, ^8^ Herein is described the case of a patient with symptoms that appear to be solely due to the loss of intraventricular left ventricular (LV) synchrony without any discernable contribution from AV dyssynchrony.

## Case report

The patient is a 70-year-old woman who initially presented to an outside hospital with recurrent near syncope. While on telemetry, she was noted to have episodes of Mobitz type 2 second-degree AV block and complete heart block correlating with symptoms. She was transferred to our facility, where echocardiography demonstrated normal LV systolic function with a left ventricular ejection fraction (LVEF) of 65%–70%, grade I diastolic dysfunction, and no significant valvular abnormalities. She underwent an uncomplicated placement of an Abbott PM2272 Assurity MRI (Abbott Laboratories, Lake Bluff, IL) dual-chamber pacemaker with Abbott 2088TC/46 and 2088TC/52 (Abbott Laboratories, Lake Bluff, IL) atrial and ventricular leads, respectively, with the latter placed at a midseptal location within the right ventricle. Atrial and ventricular sensing were excellent at >5.0 mV and >12.0 mV, respectively, as were pacing thresholds of 0.625 @ 0.4 ms and 0.75 @ 0.4 ms, respectively. Sensed and paced AV delays were initially programmed to 170 and 200 ms, respectively.

Upon return to the office at 1 week postdischarge, she complained of new-onset dyspnea, a persistent “fullness” in her chest, generalized fatigue and weakness, and exercise intolerance. Device interrogation demonstrated appropriate function, with a ventricular pacing burden of >99% but with underlying 1:1 AV conduction and a prolonged AV delay of 230 ms. Notably, she was able to immediately recognize when ventricular pacing was disabled or re-enabled, reporting resolution or recurrence of her chest “fullness” within moments of ventricular pacing being turned off or on, respectively, despite being blinded to device programming. Ventricular Intrinsic Preference was enabled; however, at follow-up a week later she reported ongoing intermittent symptoms, with her device revealing a ventricular pacing burden of 64% owing to intermittent heart block.

**Figure 1:**
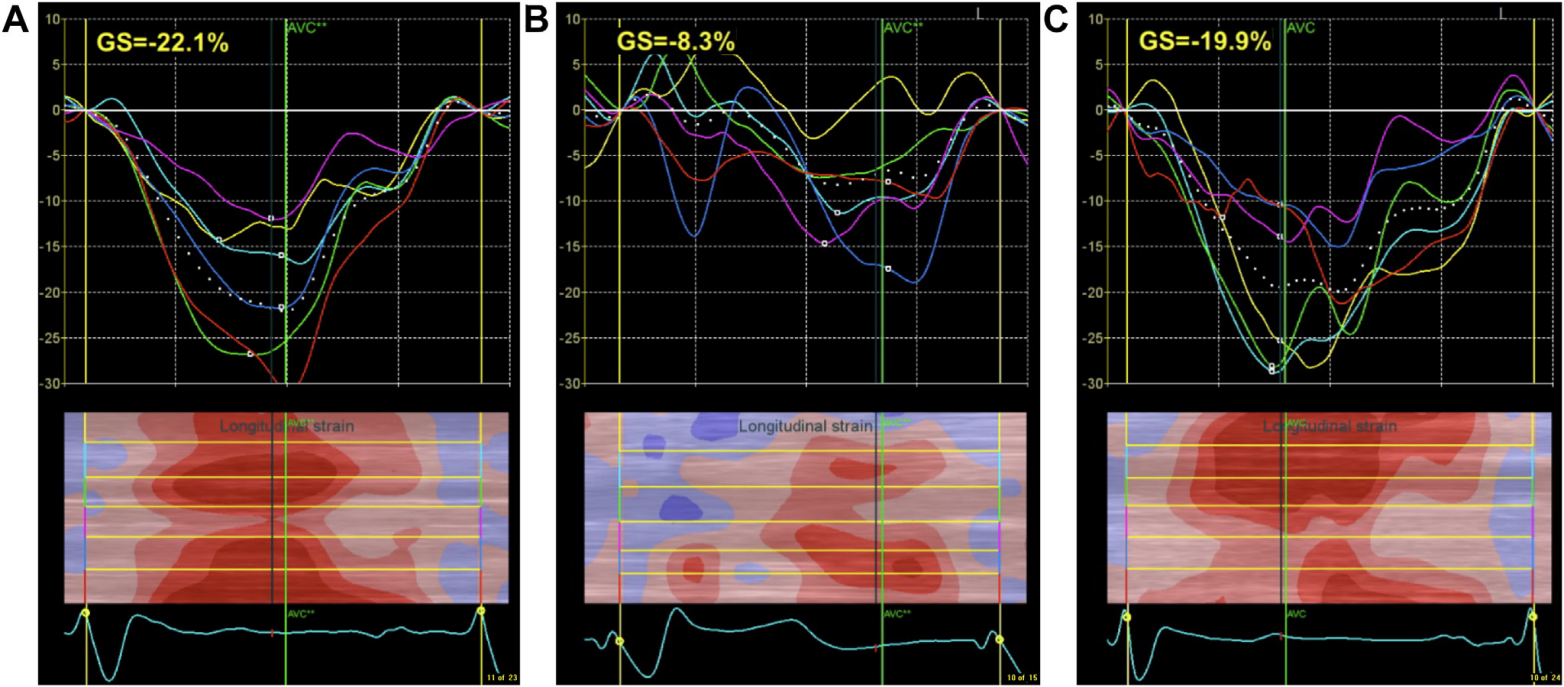
**A:** Baseline global longitudinal strain measured at -22.1% by speckle-tracking imaging during intrinsic atrioventricular conduction. **B:** Decline in longitudinal strain to -8.3% during right ventricular–only pacing. **C:** Normalization of global longitudinal strain to -19.9% with cardiac resynchronization therapy.

Further evaluation at a time when the patient exhibited intact AV conduction revealed symptomatic RV pacing across a broad range of AV delays from 120 ms to just short of her intrinsic AV conduction time. Echocardiography with speckle-tracking imaging (STI) was performed during intrinsic conduction as well as during RV pacing (Figure 1A and 1B). As compared to intrinsic conduction, RV pacing was associated with the development of lateral and apical dyskinesis, with a significant decline in global longitudinal strain from -22.1% to -8.3%.

The patient was brought back to the electrophysiology lab at 5 weeks after her original device implant, where an attempt was made to perform conduction system pacing by placing a Medtronic 3830/69 (Medtronic, Inc, Minneapolis, MN) left bundle branch area pacing lead via a C315-HIS fixed curve sheath (Medtronic, Inc, Minneapolis, MN); unfortunately, despite multiple attempts, a location yielding a suitable QRS morphology and adequate sensing/pacing thresholds could not be identified. A decision was made to upgrade to a cardiac resynchronization therapy (CRT) pacemaker system, and an Abbott 1458QL/86 (Abbott Laboratories, Lake Bluff, IL) lead was subsequently placed into a posterolateral branch of the coronary sinus, with the pulse generator being concurrently upgraded to an Abbott PM3562 Quadra Allure MP CRT-P (Abbott Laboratories, Lake Bluff, IL). The device was programmed to DDDR 60/120, with QuickOpt™-determined sensed and paced AV delays of 120 and 170 ms, respectively, and ventricular pacing set to RV first by 45 ms relative to LV. Intraoperative STI demonstrated an improvement in global longitudinal strain from -8.3% with RV-only pacing to -19.9% with CRT (Figure 1C). Immediately following her procedure and on subsequent follow-up through 1 year, the patient reported complete resolution of all symptoms.

## Discussion

Although AV dyssynchrony has been associated with a diverse and often nonspecific constellation of symptoms (eg, dyspnea, fatigue, and exercise intolerance) often referred to as the pacemaker syndrome, the potential of isolated LV dyssynchrony to cause such symptoms has not been well explored despite the well-known adverse chronic effects of RV pacing.^3^^,^^4^ A challenge in identifying the cause of such nonspecific symptoms at the individual patient level is that high-grade AV block is often nontransient and irreversible, unlike in the patient presented herein, who was able to serve as her own control for assessing the effects of RV pacing on symptoms, as her AV block was intermittent.

The exact mechanisms by which LV dyssynchrony may cause symptoms in the setting of a preserved ejection fraction are unclear, although several studies have explored the hemodynamic effects of LV dyssynchrony and may provide some insights into this question. Rosenqvist and colleagues^6^ used radionuclide and Doppler echocardiographic evaluation to demonstrate regional and global impairment of LV systolic function with DDD and VVI pacing modes as compared to AAI pacing in patients with and without heart failure, both at rest and during exercise. Similarly, Bordachar and colleagues^7^ demonstrated that echocardiographic parameters of LV dyssynchrony were highly correlated with decreases in cardiac output and worsening mitral regurgitation, albeit in patients with heart failure and a baseline LVEF <40%. Delgado and colleagues^8^ used STI to demonstrate an impairment in LV longitudinal shortening with RV-only pacing as compared to intrinsic AV conduction in patients with structurally normal hearts, as seen with this patient. Morris and colleagues^9^ subsequently examined patients with an LVEF >50% and symptomatic diastolic dysfunction; they found that LV mechanical dyssynchrony as assessed by STI resulted in elevated LV filling pressures and worsening functional capacity during exercise, and speculated a causal relationship to symptomatic heart failure with preserved ejection fraction (HFpEF).^9^ Indeed, it is possible that this patient suffered from subclinical HFpEF that was unmasked by the LV dyssynchrony induced by RV-only pacing, with subsequent control of symptoms by correction of the LV dyssynchrony via CRT. Regardless of the mechanism behind symptoms, there are currently no guidelines that specifically address the management of patients with a preserved LVEF and AV synchrony who experience symptomatic RV-only pacing.

The patient presented herein had no class I or IIa indications for cardiac physiologic pacing (CPP) via biventricular pacing, His bundle pacing, or left bundle branch area pacing given her preserved LVEF, although the recent 2023 HRS/APHRS/LAHRS Guideline on Cardiac Physiologic Pacing for the Avoidance and Mitigation of Heart Failure does give CPP in this setting a class IIb indication, albeit for the purpose of reducing the risk developing heart failure given her high anticipated ventricular pacing burden.^10^ This case is representative of a patient group not explicitly addressed by current guidelines: namely, those experiencing symptomatic LV dyssynchrony resulting from RV-only pacing in whom correction of LV dyssynchrony via CPP may provide relief. Further studies are needed to assess the generalizability of CRT and conduction system pacing as therapeutic options in such patients.

## Conclusion

Isolated LV dyssynchrony resulting from RV-only pacing may result in acute symptoms typically associated with pacemaker syndrome even in the absence of AV dyssynchrony or a depressed LVEF, perhaps by unmasking subclinical HFpEF. A subset of such patients with symptoms and LV dyssynchrony documented by STI may benefit from CPP.

## Disclosures:

No conflicts to disclose.
